# Incidence and distribution of contralateral lymph node metastasis associated with human papillomavirus-related oropharyngeal squamous cell carcinoma

**DOI:** 10.1017/S0022215124000719

**Published:** 2024-09

**Authors:** Belen Kornfeld, Lee Kyang, Ahmed Taha, Rachael McCloy, Vanessa Chin, Brett Leavers, Peter Floros, Peter Earls, Dion Forstner, Alfred Addison, Julia Crawford, Richard Gallagher

**Affiliations:** 1 Department of Otolaryngology, Head and Neck Surgery, St Vincent's Hospital, Sydney, Australia; 2 School of Medicine, Notre Dame University, Sydney, Australia; 3 The Garvan Institute of Medical Research, Darlinghurst, NSW, Australia; 4 The Kinghorn Cancer Centre, Darlinghurst, NSW, Australia; 5 Department of Anatomical Pathology, St Vincent's Hospital, Sydney, Australia; 6 Department Radiation Oncology, St Vincent's Hospital, Sydney, Australia; 7 GenesisCare, Darlinghurst, NSW, Australia

**Keywords:** Oropharyngeal neoplasms, human papillomavirus viruses, robotic surgical procedures, neck dissection, carcinoma, squamous cell

## Abstract

**Objective:**

To analyse the rate of contralateral nodal metastasis in human papillomavirus (HPV)-associated oropharyngeal carcinoma and identify the patient cohorts that would benefit from bilateral neck treatment.

**Methods:**

A retrospective cohort review was performed on 110 HPV-positive oropharyngeal carcinoma patients who underwent transoral robotic surgery and bilateral neck dissections from 2012 to 2022. The primary outcome was to investigate the pathological incidence and location of contralateral neck node metastasis.

**Results:**

The contralateral nodal disease rate was 12.7 per cent (*n* = 14), of which 2 patients (2 per cent) were occult findings, with comparable results between tongue base and tonsil sub-groups. The most commonly involved contralateral nodal station was level II (11 of 110 patients, 10 per cent). The presence of extra-nodal extension and multiple ipsilateral positive nodes was associated with increased risk of contralateral nodal disease.

**Conclusion:**

The incidence of contralateral nodal and occult disease in the studied cohort is low. The characteristics of patients who may benefit from bilateral neck treatment were demonstrated.

## Introduction

Human papillomavirus (HPV) infection has emerged as the leading cause of oropharyngeal squamous cell carcinoma, behaving in a clinically and biologically distinct manner compared with traditional smoking- or alcohol-related oropharyngeal carcinoma.^1–3^ It confers a superior prognosis, response to chemoradiotherapy and significant overall survival advantage.^4^ The eighth edition of the American Joint Committee on Cancer classification has separated this subtype out into its own tumour–node–metastasis (TNM) classification in relation to this.^5^

Human papillomavirus-associated oropharyngeal carcinoma has been shown to metastasise early in its course to cervical lymph nodes, often in increased number and extent, and irrespective of the primary tumour size.^6^ However, this has different prognostic implications compared with the HPV-negative population.^4,7^

Current management for early-stage HPV-associated oropharyngeal carcinoma aims for single or dual-modality treatment with either primary transoral robotic surgery and neck dissection, or radiotherapy (RT).^8^ Chemotherapy is advised in addition to primary RT if there is evidence of neck nodal metastasis.^7^ Adjuvant therapy after surgery is recommended for unfavourable characteristics on histopathology such as evidence of perineural invasion, lymphovascular invasion, extra-nodal extension or persistent disease.^1,8^ The extent of treatment delivered to the neck, regardless of the chosen modality (surgery or RT), has evolved with time and is not currently stratified for HPV status. In current practice, well-lateralised tumours undergo unilateral neck treatment.^1^

Bilateral neck treatment is recommended for clinical or radiological evidence of contralateral disease and for primary tumours extending to, or close to, the midline (particularly base of tongue tumours). This is based on concern for contralateral spread through crossing lymph node channels.^1,7^ However, the treatment of the clinically node-negative contralateral neck in patients with HPV-positive disease remains controversial. Few publications have attempted to address this issue, with no consensus achieved.^9–11^

At our institution, prior to 2019 (publication of the American Society of Clinical Oncology guidelines) we specifically performed bilateral neck dissections on all patients with HPV-associated oropharyngeal carcinoma irrespective of primary disease subsite to ascertain the rate of occult contralateral disease. Hence, the aim of this study was to change clinical practice to determine if there are specific patient cohorts that will benefit from bilateral neck treatment through defining the pathological incidence and location of contralateral nodal disease (both occult and known) by primary disease subsite.

## Materials and methods

### Study design

This study received ethics approval from the St Vincent's Hospital Human Research Ethics Committee (LNR/17/SVH/282) in accordance with the ethical standards of the Declaration of Helsinki. We performed a retrospective study on all patients who underwent bilateral neck dissections with concurrent or staged transoral robotic resection of their primary tumour for HPV-associated oropharyngeal carcinoma at our tertiary medical centre between 2012 and 2022. Data were collected directly from patient medical records and through our institution's data system.

### Patients

All patients were presented at the St Vincent's Hospital head and neck cancer multidisciplinary team (MDT) in person, and the consensus of the best treatment modality was determined. All patients underwent diagnostic computer tomography (CT) neck and chest imaging and positron emission topography (PET), which assisted clinical staging. We included all early-staged patients who underwent transoral robotic surgery and bilateral neck dissection as primary treatment for biopsy-proven early-stage (T1–T2 and select T3) HPV-related oropharyngeal carcinoma. We excluded patients with a history of prior head and neck cancer, patients with unknown primary disease, patients who had had previous neck RT or chemotherapy treatments, and patients who had had their neck dissections performed at other institutions.

### Clinical and pathologic features

Tumour staging was determined according to the updated eighth edition of the staging system for malignant head and neck tumours of the American Joint Committee on Cancer.^4^ Those who were initially staged prior to 2018, using the seventh edition of the American Joint Committee on Cancer staging system, were restaged according to the eighth edition. Data collected included age at time of diagnosis, co-morbidities, sex, HPV status (by both P16 and in situ hybridisation testing), smoking status, alcohol consumption, presenting symptoms, clinical and pathologic TNM classification, margin status, number and locations of lymph nodes involved, presence of high-risk pathological features such as perineural invasion, lymphovascular invasion, extra-nodal extension and margin status.

### Treatment and follow up

Prior to the publication of the American Society of Clinical Oncology clinical guidelines (2019), all patients underwent transoral robotic surgery resection and bilateral neck dissection irrespective of the primary tumour subsite. Modified radical neck dissection (levels I–V) was performed for the clinically node positive neck and selective II–IV for the contralateral clinically confirmed (c) cN0 neck or if the ipsilateral neck was cN0. Patients with clinical evidence of bilateral nodal disease (cN2) underwent bilateral neck dissection irrespective of the primary subsite.

In 2019 our practice changed, and the extent of neck dissection is now dictated by primary subsite and proximity to the midline where patients with well-lateralised primary tumours underwent unilateral neck dissection. These patients were not included in this study.

Transoral robotic surgery was performed using Da Vinci® Surgical System Si and subsequently Xi. The tumour was resected en bloc with additional resection margins in areas of concern following macroscopic evaluation by the surgeon or from microscopic confirmation via intra-operative frozen section examination. Neck dissection was performed concurrently when the primary site was addressed or staged within a fortnight of the transoral robotic surgery resection.

Each nodal level was submitted for histopathology separately. Surgical histopathology was discussed at the St Vincent's Hospital head and neck cancer pathology MDT meeting to determine the need for adjuvant therapy. The indication for adjuvant therapy was guided by National Comprehensive Cancer Network guidelines and determined by high-risk features on histopathology, including margin involvement, perineural invasion, lymphovascular invasion and the presence of extra-nodal extension.

Adjuvant treatment included RT or RT with concurrent chemotherapy. The RT dose and fields were individualised for each patient. In cases with involved margins or extra-nodal extension, concurrent chemotherapy was used. Patients with involved margins received 64–66 Gy in 30 to 33 fractions to the primary tumour site. Involved nodal levels received 60 Gy and uninvolved levels received 54 Gy in 30 fractions. If the neck was not involved on review of the pathology, RT was provided to the primary site alone. Similarly, if the primary site had clear margins, then the primary site was spared and only the neck received adjuvant therapy if there was evidence of high-risk features.

Long-term follow up was scheduled at three-monthly intervals for the first two years, four-monthly intervals in the third year and six-monthly intervals thereafter to five years. A post-treatment PET-CT with diagnostic CT of the neck and chest was performed at three months and then yearly until five years.

### Outcomes

The primary outcome was to determine the incidence and location of contralateral neck metastases in the study cohort by histopathological analysis. Secondary outcomes included the frequency and distribution of metastases in the ipsilateral neck for each oropharyngeal subtype, clinicopathological features associated with contralateral neck metastases, overall survival and locoregional recurrence.

### Statistical analysis

All statistical analyses were performed using SPSS for Windows version 25 (IBM Corporation, New York, USA). Patient characteristics were described using frequency and descriptive analyses. Survival analyses of neck dissection were estimated using Kaplan–Meier curves and the log rank test for comparison.

Clinicopathological factors of patients with contralateral negative pathological nodes and those with positive nodes were compared using the *X*^2^ test for categorical variables. Significant factors (*p* ≤ 0.05) were further analysed using a binary logistic regression model. In the positive contralateral pathological node group, prognostic factors affecting overall survival and disease-free survival were investigated using a Cox regression model. Significant parameters (*p* < 0.10) were chosen for multivariable analysis and *p* less than 0.05 was considered statistically significant.

## Results and analysis

### Patients characteristics

Patient demographics are presented in Table 1. There were 110 patients included in the study. The majority of patients were male (*n* = 92, 83.6 per cent), non-smokers (*n* = 58, 52.7 per cent) and non-drinkers (*n* = 46, 41.8 per cent). The median age at time of diagnosis was 58.9 years (standard deviation (SD) = 8.5).

**Table 1.** Patients’ demographics and characteristics

Characteristics	Patients
Gender (*n* (%))	
– Male	92 (83.6)
– Female	18 (16.4)
Age (mean (SD); years)	58.9 (8.5)
Smoking (*n* (%))	
– Never	58 (52.7)
– Past (>100 cigarettes)	41 (37.3)
– Current	11 (10.0)
Alcohol (g/day (%))	
– None	46 (41.8)
– 1–20	27 (24.5)
– 20–40	14 (12.7)
– >40	21 (19.1)
– Unknown	2 (1.8)

SD = standard deviation

### Tumour characteristics and treatment data

In our cohort, most patients (*n* = 98, 89.1 per cent) presented with cT1–2 disease, while 12 patients (10.9 per cent) had a cT3 tumour. There were equal numbers of patients with tonsillar (*n* = 48, 43.6 per cent) and base of tongue primary (*n* = 48, 43.6 per cent) tumours, with the majority being cN1+ (*n* = 79, 71.8 per cent) at time of diagnosis. Pre-operative clinical or radiological evidence of contralateral positive lymph nodes were found in 12 patients (10.9 per cent).

Most patients (*n* = 88, 80 per cent) underwent a unilateral modified radical neck dissection and a contralateral selective neck dissection. The average total lymph node yield was 83.8 (SD = 21.5). The median number of positive lymph nodes per patient was 2.6 (SD = 3.6). The contralateral nodal disease rate was 12.7 per cent (*n* = 14). Among the 98 patients who did not have clinical evidence of contralateral nodal disease, only 2 (2 per cent) had contralateral nodal disease demonstrated on histopathology after surgery. Of these two patients, one patient had a tonsil primary and the other patient had a base of tongue primary. Both patients were T2N1 at presentation (T2N2b according to the seventh edition of the American Joint Committee on Cancer staging system). There were no patients who had a negative ipsilateral neck (*n* = 17, 15.4 per cent) with evidence of contralateral neck disease. Of those patients with contralateral nodal disease (*n* = 14), 8 of 48 (16.6 per cent) patients were in the base of tongue group, 6 of 48 (12.5 per cent) were in the tonsil group and none were in the glossotonsillar sulcus group.

High-risk pathological features were identified in 62 patients (56 per cent). Perineural invasion, lymphovascular invasion and extra-nodal extension were identified in 14 patients (12.7 per cent), 27 patients (24.5 per cent) and 21 patients (19 per cent), respectively. Adjuvant therapy was recommended based on final pathology and was indicated in less than half of patients. Additional tumour clinical and pathological characteristics are summarised in Table 2.

**Table 2.** Clinicopathological characteristics of HPV-related oropharyngeal squamous cell carcinoma undergoing transoral robotic surgery and bilateral neck dissection

Characteristics	Cases (*n* (%))
Tumour site	
– Base of tongue	48 (43.6)
– Tonsil	48 (43.6)
– Glossotonsillar sulcus	14 (12.7)
Clinical T stage	
– T1	55 (50.0)
– T2	43 (39.1)
– T3	12 (10.9)
Clinical N stage	
– N0	17 (15.4)
– N1	79 (71.8)
– N2	13 (11.8)
– N3	1 (0.9)
Pathological T stage	
– T1	45 (40.9)
– T2	59 (53.6)
– T3	6 (5.5)
Pathological N stage	
– N0	16 (14.5)
– N1	79 (71.8)
– N2	14 (12.7)
– N3	1 (0.9)
Lymph node yield	Mean (SD)
– Total	83.8 (21.5)
– Positive	2.6 (3.6)
Type of surgery	
– Bilateral MRND	6 (5.5)
– Bilateral SND	16 (14.5)
– Unilateral MRND + contralateral SND	88 (80.0)
HPV in situ hybridisation status	
– Positive	100 (90.9)
– Negative/equivocal	6 (5.5)
– Not tested	4 (3.6)
Perineural invasion	
– Yes	14 (12.7)
– No	96 (87.3)
Lymphovascular invasion	
– Yes	28 (25.5)
– No	82 (74.5)
Extra-nodal extension	
– Yes	21(19.1)
– No	89 (80.9)
Adjuvant therapy	
– Yes	50 (45.5)
– Radiotherapy	20 (18.2)
– Chemoradiotherapy	30 (27.3)
– No	60 (54.5)

HPV = human papillomavirus; SD = standard deviation; MRND = modified radical neck dissection; SND = selective neck dissection

### Location of nodal metastasis

Table 3 illustrates the laterality and location of nodal neck disease based on primary subsite. The most commonly involved contralateral nodal station was level II (11 of 110 patients, 10 per cent), followed by level III (4 of 110 patients, 3.6 per cent), level IV (2 of 110 patients, 1.8 per cent), level Va (2 of 110 patients, 1.8 per cent) and level Vb (1 of 110 patients, 0.9 per cent). For all subsites, level II (particularly IIa) was the most frequently involved nodal level (>70 per cent) in the ipsilateral neck for all oropharyngeal subsites. Level III was involved in 31.3 per cent of lingual tonsil tumours, 22.9 per cent of palatine tonsil tumours and only 7.1 per cent of glossotonsillar sulcus tumours. Level IV was less commonly involved (8.3 per cent base of tongue, 10.4 per cent tonsil, 7.1 per cent glossotonsillar sulcus). Level I involvement was infrequent, with only 4.2 per cent of tonsil primaries developing nodal metastasis in this level and none for base of tongue and glossotonsillar sulcus groups. Level V (including Va and Vb) was less commonly involved, occurring in less than 10 per cent in tonsil, less than 5 per cent in base of tongue and never in glossotonsillar sulcus tumours. Table 4 compares the distribution of nodal disease on both sides of the neck by clinical N stage.

**Table 3.** Nodal metastasis distribution based on primary subsite on histopathology

Location	Positive nodes (%)		
	Base of tongue	Tonsil	Glossotonsillar sulcus
Proportion of neck dissection with metastases			
Nil	10.4	16.7	21.4
Ipsilateral	72.9	70.8	78.6
Bilateral	16.7	12.5	0
Neck side and level			
Ipsilateral neck			
– Level I	0	4.2	0
– Level II	4.2	6.3	7.1
– Level IIa	79.2	79.2	57.1
– Level IIb	12.5	22.9	7.1
– Level III	29.2	22.9	7.1
– Level IV	4.2	8.3	7.1
– Level Va	4.2	8.3	0
– Level Vb	2.1	6.3	0
Contralateral neck			
– Level I	0	0	0
– Level II	2.1	0	0
– Level IIa	12.5	12.5	0
– Level IIb	4.2	6.3	0
– Level III	6.3	4.2	0
– Level IV	4.2	2.1	0
– Level Va	0	2.1	0
– Level Vb	2.1	0	0

**Table 4.** Side and level distribution of neck metastases by clinically confirmed nodal (cN+) classification

Neck side and level	Positive nodes (%)			
	cN0	cN1	cN2	cN3
Ipsilateral neck				
– Level I	0	97.1	25	0
– Level II	83.3	92.9	100	0
– Level IIa	66.7	14.3	100	100
– Level IIb	50	22.9	25	0
– Level III	16.7	5.7	50	0
– Level IV	0	1.4	25	0
– Level Va	0	1.4	25	0
– Level Vb	0	25	25	0
Contralateral neck				
– Level I	0	25	0	0
– Level II	0	100	100	0
– Level IIa	0	100	90	0
– Level IIb	0	50	40	0
– Level III	0	75	50	0
– Level IV	0	50	30	0
– Level Va	0	25	30	0
– Level Vb	0	0	100	0

### Variables associated with contralateral neck disease

Table 5 highlights the results of univariable and multivariate analysis of clinicopathological factors associated with contralateral nodal metastasis in HPV-related oropharyngeal carcinoma. Univariate logistic regression analysis revealed that pathologically confirmed (p) pT3, pN2–3, the presence of multiple ipsilateral positive nodes, extra-nodal extension, and perineural invasion were associated with increased risk contralateral neck metastases (*p* < 0.05). However, on multivariable analysis, only extra-nodal extension and multiple ipsilateral positive nodes remained a statistically significantly predictive on contralateral nodal disease.

**Table 5.** Characteristics and predictive factors for contralateral disease

Clinical and pathological factors	Contralateral pN0 (*n* = 96)	Contralateral pN+ (*n* = 14)	*p* value	Multivariate analysis, OR (95% CI, *p* value)
Gender (*n* (%))				
– Female	18 (100)	0 (0)		
– Male	78 (84.8)	14 (15.2)	0.08	
Tumour location (*n* (%))				
– Base of tongue	40 (83.3)	8 (16.7)		
– Tonsil	42 (87.5)	6 (12.5)		
– Glossotonsillar sulcus	14 (100)	0 (0)		
Smoking (*n* (%))				
– Never	51 (87.9)	7 (12.1)	0.85	
– Current	9 (81.8)	2 (18.2)		
– Past	36 (87.8)	5 (11.9)		
Alcohol (*n* (%))				
– None	42 (91.3)	4 (8.7)	0.58	
– 0–20 g/day	23 (85.2)	4 (14.8)		
– 20–40 g/day	11 (78.6)	3 (21.4)		
– >40 g/day	19 (90.5)	2 (9.5)		
Clinical T stage (*n* (%))				
– T1–T2	85 (86.7)	13 (13.3)	0.63	
– T3	11 (91.7)	1 (8.3)		
Number of ipsilateral node involvement (*n* (%))				
– Single or none	73 (94.8)	4 (5.2)	<0.05	4.99 (1.15-21.6, *p* < 0.05)
– Multiple	23 (69.7)	10 (30.3)		
Pathological T stage (*n* (%))				
– T1–T2	93 (89.4)	11 (10.6)	<0.05	6.08 (0.83–44.65, *p* = 0.08)
– T3	3 (50.0)	3 (50.0)		
Perineural invasion (*n* (%))				
– Yes	9 (64.3)	5 (35.7)	<0.05	2.62 (0.57–12.08, *p* = 0.22)
– No	86 (90.5)	9 (9.5)		
Lymphovascular invasion (*n* (%))				
– Yes	20 (74.1)	7 (25.9)	<0.05	1.90 (0.48–7.44, *p* = 0.36)
– No	75 (91.5)	7 (8.5)		
Extra-nodal extension (*n* (%))				
– Yes	13 (61.9)	8 (38.1)	<0.05	6.658 (1.76–24.60, *p* < 0.05)
– No	83 (93.3)	6 (6.7)		

p = pathologically confirmed; OR = odds ratio; CI = confidence interval

### Recurrence and overall survival

In total, 22 patients (20 per cent) experienced disease recurrence. The locoregional recurrence rate was 11.8 per cent (*n* = 13). Most recurrences (18 of 22 patients, 81.8 per cent) were observed within the first 2 years post-treatment. Half of the recurrences (11 of 22 patients) were successfully salvaged through varying combinations of surgery and systemic treatments. Further details on recurrence based on clinical and pathological nodal status can be found in Table 6.

**Table 6.** Recurrence based on contralateral neck clinically confirmed (c) and pathologically confirmed (p) findings

Recurrence	Patient group (*n* (%))		
	cN2/pN2 (*n* = 12)	cN0–1/pN2 (*n* = 2)	cN0–1/pN0–1 (*n* = 96)
No recurrence	7 (58.3)	1 (50)	80 (83.3)
Locoregional	1 (8.3)	0	12 (12.5)
Locoregional + metastatic	2 (16.7)	1 (50)	2 (2.1)
Distant metastatic disease	2 (16.7)	0	2 (2.1)

**Figure 1.**
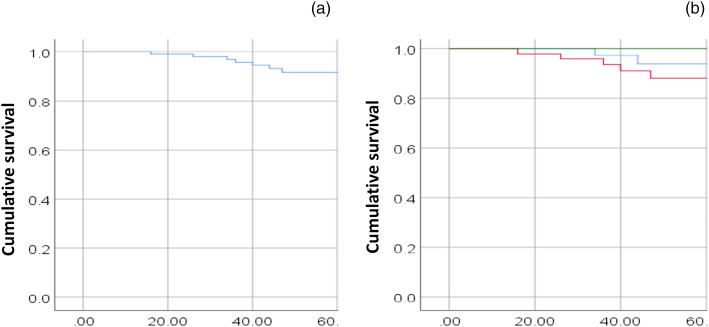
Kaplan–Meier graphs of (a) overall survival and (b) overall survival based on primary tumour subsite for patients with human papillomavirus-associated oropharyngeal squamous cell carcinoma undergoing transoral robotic surgery and bilateral neck dissection. Green line = glossotonsillar sulcus; blue line = base of tongue; red = tonsil

The 3- and 5-year recurrence-free survival rates were 81.7 and 80.2 per cent, respectively, while overall survival was 95.8 per cent at 3 years and 91.7 per cent at 5 years. When analysing overall survival and recurrence-free survival in relation to the presence of contralateral neck disease, no statistically significant difference was observed (*p* > 0.05). Figure 1 demonstrates the overall survival for the cohort and when subdivided by primary tumour site.

On multivariate analyses, no significant prognostic factors influencing overall survival and recurrence-free survival were identified, including gender, alcohol, tumour locations, smoking status, clinical T stage, pathological T stage, perineural invasion, lymphovascular invasion, extra-nodal extension and adjuvant therapy. However, when comparing patient cohorts with only ipsilateral involved lymph nodes versus those with contralateral nodal disease, multivariate analysis revealed extra-nodal extension, perineural invasion and pathological T stage were prognostic factors for overall survival in those with contralateral nodal involvement. In contrast, only extra-nodal extension and perineural invasion were identified as poor prognostic factors for recurrence-free survival (refer to Table 7).

**Table 7.** Independent prognostic factors influencing overall survival and recurrence-free survival for patients with positive contralateral node on multivariate analyses

Factors	Overall survival		Recurrence-free survival	
	HR (95% CI)	*p* value	HR (95% CI)	*p* value
Extra-nodal extension	17.49 (4.14-73.93)	<0.05	18.48 (4.52-75.61)	<0.05
Perineural invasion	9.36 (2.14-40.83)	<0.05	5.06 (1.31-19.53)	<0.05
pT stage	9.98 (1.56-63.74)	<0.05	3.24 (0.21-3.40)	0.17
Lymphovascular invasion	0.31 (0.06-1.56)	0.15	0.85 (0.21-3.40)	0.82

HR = hazard ratio; CI = confidence interval

## Discussion

This study aimed to investigate the rate and location of occult contralateral nodal metastasis in surgically managed HPV-related oropharyngeal carcinoma. The contralateral nodal disease rate in the study cohort was 12.7 per cent (*n* = 14), with a marginal difference between the base of tongue and tonsil groups (*n* = 2 patients, base of tongue 16.6 per cent *vs* tonsil 12.5 per cent). Our study is the first to separate patients with glossotonsillar sulcus tumours as a separate primary subsite (*n* = 14), and none showed evidence of contralateral nodal disease.

Only 2 per cent of patients (*n* = 2) had occult contralateral disease on histopathology. This was comparable between the base of tongue and tonsil primary tumour groups, and both patients had presented with multiple clinically evident ipsilateral lymph nodes. There have been few studies that have investigated the incidence and location of contralateral neck disease in HPV-associated oropharyngeal carcinoma, with varying methodologies and heterogeneous results.^2,12–19^ Smith *et al*. conducted a study similar to ours that examined the incidence of contralateral lymph node metastasis in 120 patients with early stage (T1–T2) HPV-related oropharyngeal carcinoma who underwent transoral robotic surgery and bilateral neck dissection. Their study demonstrated a contralateral nodal disease rate similar to ours of 9 of 120 patients (7.5 per cent).^7^

McMullen investigated a smaller sample size of only 32 patients using similar methodology and found a similarly low occult contralateral nodal rate of the p16+ subgroup of 5 per cent (1 of 20 patients).^20^ A retrospective series conducted by Last *et al*. on HPV-related base of tongue tumours undergoing transoral robotic surgery and bilateral neck dissection found a significantly higher occult contralateral nodal disease rate of 21.4 per cent (15 of 70 patients) on pathological assessment.^17^ It is unclear however, if all patients underwent diagnostic CT and/or PET scanning prior to surgery, which may have underestimated the known contralateral disease rate.

Previous studies have estimated HPV-related oropharyngeal carcinoma contralateral nodal involvement to range between 9 and 22 per cent.^17,18^ However, these studies were limited by identifying contralateral nodal disease in non-surgically treated patients and by physical examination and/or imaging only. Such figures have encouraged many institutions to treat both sides of the neck without convincing evidence. Surgical neck dissection with histopathological analysis is the most accurate method of determining occult nodal disease burden and is this foundation of our study.

De-intensification strategies, particularly in HPV-related disease, are at the forefront of research, with the aim of minimising potential complications and toxicity, and improving quality of life.^21^ Defining which patients would benefit from bilateral neck treatment and the extent of neck treatment required regardless of treatment modality is imperative to minimise toxicity and adverse effects. This is of even higher importance given that this patient cohort is generally younger and has a longer post-treatment life expectancy than those with HPV-negative disease.

In our cohort, there was a significant number of patients (*n* = 30, 27.3 per cent) who required chemotherapy in addition to RT as adjuvant treatment. This was largely due to extra-nodal extension (*n* = 21, 19 per cent) demonstrated on final histopathology that was not evident on prior imaging. This highlights the difficulty in risk-stratifying patients based on clinical examination and radiology when aiming for dual-modality treatment. Our findings indicate that the presence of extra-nodal extension and multiple ipsilateral positive nodes is the most significant predictive factor for contralateral disease involvement. The presence of extra-nodal extension has not yet been acknowledged as an important prognostic marker for risk of contralateral nodal disease in the HPV-related oropharyngeal carcinoma cohort in existing literature, whereas the presence of multiple ipsilateral nodes has been established.^7,13,14^

Previous studies have suggested without convincing evidence that tumour lateralisation, proximity to midline, and advanced T stage are the most crucial prognostic indicators, but this does not appear to be HPV-specific.^12,13,15,18^ Based on our data, when considering bilateral neck treatment, in the contralateral neck, level IIa was the most frequently involved station (12.5 per cent in both the base of tongue and tonsil groups) while the risk of additional nodal station involvement was less than 7 per cent. This may assist in rationalising the extent of neck dissection required for the contralateral neck when considering bilateral neck dissection. Our study's favourable overall survival and recurrence-free survival rates, along with the lack of significant prognostic implications of contralateral neck disease affecting survival rates, further supports the needs for de-escalation efforts.

Our study contributes to the expanding literature that aims to characterise patterns of nodal metastasis in HPV-associated oropharyngeal carcinoma and its subsites to determine the necessary extent of neck dissection. Among surgical risks, the potential damage to neurovascular structures, particularly the spinal accessory nerve during level V dissection, poses the greatest long-term concern and can result in weakened shoulder abduction.

In all subsites of the oropharynx, level II (particularly level IIa) was the most commonly involved (>70 per cent) in the ipsilateral neck, followed by levels III and IV. Level V (including Va and Vb) showed infrequent involvement, occurring in less than 10 per cent of palatine tonsil cases, less than 5 per cent of lingual tonsil cases and never in glossotonsillar sulcus tumours. This supports previous published studies reporting low rates of level V involvement ranging between 3.4 and 9.9 per cent, the higher rate associated with increasing N stage.^2,22,23^ The major determinant of level V positivity is the presence of additional lymph node level involvement, which is supported by our data.^22,23^ However, the available studies vary in methodology and often lack comparisons between primary subsite or HPV-specific characterisation. Nevertheless, it is important to consider the findings of Bauwens and Kato *et al*., whose studies compared HPV-positive and -negative cohorts, and found no significant difference in nodal metastatic patterns between these groups.^14,18^

Our study was primarily limited by its retrospective design, which inherently will have introduced variability in the analysis and documentation of pathologic variables. However, given our study was conducted at a single institution with two surgeons using similar techniques with regard to en bloc transoral robotic surgery resection, neck dissection and histopathological analysis, there is an element of reciprocity.

- Treatment of the clinically node-negative contralateral neck in patients with human papillomavirus (HPV)-positive oropharyngeal squamous cell carcinoma (oropharyngeal carcinoma) disease remains controversial
- In current practice, well-lateralised tumours undergo unilateral neck treatment and bilateral neck treatment is recommended for clinical or radiological evidence of contralateral disease, and for primary tumours extending to, or close to the midline (particularly tongue base tumours)
- In this study of HPV-associated oropharyngeal carcinoma patients, a clinically negative contralateral neck is most likely to be a pathologically negative neck as occult contralateral nodal disease is a rare occurrence (2 per cent)
- The incidence of bilateral nodal metastasis in HPV-related oropharyngeal carcinoma is 12.7 per cent (14 of 110) with no significant difference between tongue base and tonsil tumours
- Patients with multiple ipsilateral neck nodes (clinically node positive) or evidence of extra-nodal extension may benefit from bilateral neck treatment
- Patients with glossotonsillar sulcus primaries and the clinically confirmed N0 neck are appropriate for ipsilateral neck treatment
- There is a high overall survival rate (91.7 per cent) for surgically treated HPV-related oropharyngeal carcinoma irrespective of bilateral neck disease or recurrence

Although this study is one of the largest HPV-specific cohort studies in this topic, when considering both primary subsite and incidence of contralateral nodal disease, this further reduces the sample size of the population when analysing nodal involvement. Furthermore, the risk of selection bias exists because our cohort represents a specific group of patients treated at a single tertiary centre. Another limitation is the lack of data on primary tumour proximity to the midline, which would have been valuable for comparing our results with existing literature. Despite these limitations, our study provides insight into specific patient cohorts that may benefit from bilateral neck treatment and can guide the extent of neck treatment and de-intensification strategies.

## Conclusion

The contralateral lymph node metastasis rate in HPV-associated oropharyngeal carcinoma is consistently low (12.7 per cent), with rare occurrences of occult contralateral disease (2 per cent). Our study has demonstrated that a clinically negative contralateral neck is likely to be also pathologically negative. We can now offer these patients ipsilateral neck treatment with more certainty and an understanding that occult contralateral metastatic disease can occur rarely, and this outcome should be carefully monitored for in the follow-up period.

Patients with clinical or radiological evidence of bilateral nodal disease should undergo bilateral neck treatment with the aim of using dual-modality treatment. Patients with evidence of extra-nodal extension or multiple ipsilateral lymph nodes may benefit from bilateral neck treatment. For those receiving bilateral neck treatment, level II in the contralateral neck should be treated, with further prospective research required to confidently de-escalate other nodal stations. This study also demonstrated high overall survival rates (91.7 per cent) for surgically treated HPV-related oropharyngeal carcinoma irrespective of bilateral neck disease or recurrence.
